# Convulsive behaviors of spontaneous recurrent seizures in a mouse model of extended hippocampal kindling

**DOI:** 10.3389/fnbeh.2022.1076718

**Published:** 2022-12-23

**Authors:** Anya Zahra, Yuqing Sun, Nancy Aloysius, Liang Zhang

**Affiliations:** ^1^Krembil Research Institute, University Health Network, Toronto, ON, Canada; ^2^Division of Neurology, Department of Medicine, University of Toronto, Toronto, ON, Canada

**Keywords:** hippocampus, kindling, EEG, spontaneous recurrent seizures, epilepsy, mice

## Abstract

Growing studies indicate that vigilance states and circadian rhythms can influence seizure occurrence in patients with epilepsy and rodent models of epilepsy. Electrical kindling, referred to brief, repeated stimulations of a limbic structure, is a commonly used model of temporal lobe epilepsy. Kindling *via* the classic protocol lasting a few weeks does not generally induce spontaneous recurrent seizures (SRS), but extended kindling that applies over the course of a few months has shown to induce SRS in several animal species. Kindling-induced SRS in monkeys and cats were observed mainly during resting wakefulness or sleep, but the behavioral activities associated with SRS in rodent models of extended kindling remain unknown. We aimed to add information in this area using a mouse model of extended hippocampal kindling. Middle-aged C57 black mice experienced ≥80 hippocampal stimulations (delivered twice daily) and then underwent continuous 24 h electroencephalography (EEG)-video monitoring for SRS detection. SRS were recognized by EEG discharges and associated motor seizures. The five stages of the modified Racine scale for mice were used to score motor seizure severities. Seizure-preceding behaviors were assessed in a 3 min period prior to seizure onset and categorized as active and inactive. Three main observations emerged from the present analysis. (1) SRS were found to predominantly manifest as generalized (stage 3–5) motor seizures in association with tail erection or Straub tail. (2) SRS occurrences were not significantly altered by the light on/off cycle. (3) Generalized (stage 3–5) motor seizures were mainly preceded by inactive behaviors such as immobility, standing still, or apparent sleep without evident volitional movement. Considering deeper subcortical structures implicated in genesis of tail erection in other seizure models, we postulate that genesis of generalized motor seizures in extended kindled mice may involve deeper subcortical structures. Our present data together with previous findings from post-status epilepticus models support the notion that ambient cage behaviors are strong influencing factors of SRS occurrence in rodent models of temporal lobe epilepsy.

## Introduction

Epilepsy is a chronic neurological disorder characterized by unprovoked seizures or spontaneous recurrent seizures (SRS) and comorbidities. Growing evidence indicates that epileptic seizures occur with different temporal patterns and that vigilance states and circadian variations can both influence seizure occurrence and epileptic activity [see reviews by Khan et al. (2018), Frauscher and Gotman (2019), Leite Góes Gitai et al. (2019), Grigg-Damberger and Foldvary-Schaefer (2021), Karoly et al. (2021)]. For example, in patients with frontal lobe epilepsy or sleep-related hyper motor epilepsy, seizures were found to occur predominantly during sleep primarily in non-rapid eye movement sleep (Tinuper et al., 2016). In patients with temporal lobe epilepsy, about 60% of seizures were found to occur in the daytime period, with incidence peaking in the afternoon (Quigg et al., 1998). Other studies have reported a bimodal distribution of seizure occurrence in patients with temporal lobe epilepsy, with a primary incidence peak in the late afternoon and a secondary peak in the morning (Karafin et al., 2010; Passarelli and Castro, 2015; Nzwalo et al., 2016; Spencer et al., 2016). A current view is that further understanding of the temporal patterns and underlying mechanisms of epileptic seizures may enable the development of novel treatment options for individuals with epilepsy (Khan et al., 2018; Frauscher and Gotman, 2019; Leite Góes Gitai et al., 2019; Grigg-Damberger and Foldvary-Schaefer, 2021; Karoly et al., 2021).

Temporal patterns in seizure occurrence are also recognizable in rodent models of temporal lobe epilepsy. In post-status epilepticus models, chronic SRS developed after a latent period following an acute episode of status epilepticus. When monitored in the experimental setting with a 12 h light on/off cycle (generally with light-on 7–8 a.m. to 7–8 p.m.), SRS were more frequently observed during the light on than during the light off period (Cavalheiro et al., 1991; Bertram and Cornett, 1994; Quigg et al., 1998; Arida et al., 1999; Hellier and Dudek, 1999; Goffin et al., 2007; Raedt et al., 2008; Tchekalarova et al., 2011; Pitsch et al., 2017). Moreover, SRS were found to arise mainly during periods of inactivity with little to no volitional movement (Hellier and Dudek, 1999; Pitsch et al., 2017).

Electrical kindling, referred to brief and repeated stimulation of a limbic structure, is a commonly used model of temporal lobe epilepsy (Gorter et al., 2001; Löscher, 2016; Sutula and Kotloski, 2017). While kindling through the classic or standard protocol with once or twice daily stimulations for a few weeks does not generally induce SRS (see however, Haas et al., 1992). Extended kindling that applies once or twice daily stimulations over the course of a few months can induce SRS. Specifically, kindling-induced SRS have been observed in monkeys (Wada et al., 1975; Wada and Osawa, 1976), dogs (Wauquier et al., 1979), cats (Wada et al., 1974; Gotman, 1984; Shouse et al., 1990; Hiyoshi et al., 1993), rats (Pinel and Rovner, 1978a,b; Milgram et al., 1995; Michalakis et al., 1998; Sayin et al., 2003; Brandt et al., 2004), and mice (Song et al., 2018; Liu et al., 2021). Unlike the post-status epilepticus models (Dudek and Staley, 2017; Gorter and van Vliet, 2017; Henshall, 2017; Kelly and Coulter, 2017), SRS development in extended kindled animals is not associated with initial status epilepticus. As such, the extended kindling model may complement the post-status epilepticus and other models (Walker et al., 2017) to explore diverse epileptogenic processes relevant to human temporal lobe epilepsy (Engel, 1996; Ferlazzo et al., 2016) and to explore antiepileptic manipulations.

In kindled monkeys and cats, SRS were observed mainly during resting wakefulness or sleep (Wada et al., 1975; Hiyoshi et al., 1993). However, it is unclear whether similar behavioral and circadian patterns are characteristic of SRS in kindled rats and mice. Moreover, the severity and behavioral relevance of spontaneous motor seizures remain to be quantitatively analyzed in rodent models of extended kindling. Our lab has established a protocol of extended hippocampal kindling in mice. Previous works of our lab examined responses of SRS to some of the clinically used antiepileptic drugs (Song et al., 2018), clustering expression of SRS (Liu and Zhang, 2021), spatial memory impairment (Liu et al., 2019), and regional expression and waveforms of spontaneous EEG discharges (Liu et al., 2021) in the mouse model of extended hippocampal kindling. The aim of the present study was to continue characterize SRS in kindled mice, with a particular focus on motor seizure severity and seizure-preceding behaviors.

## Materials and methods

### Animals

We conducted experiments in C57 black mice (C57BL/6N; Charles River Laboratory, Saint-Constant, Quebec, Canada). All mice were housed in standard cages with food and water *ad libitum* and kept in a local vivarium that was maintained at room temperatures between 22 and 23°C and with a 12 h light-on/off cycle (light-on starting at 6:00 a.m.). Hippocampal kindling was performed in middle-aged male mice of 11–13 months-old mice. This is to model new-onset seizures like those seen in adult and aging humans (Ferlazzo et al., 2016), while minimizing the potential occurrence of health-related complications such as skin lesions, ear infections, and tumors that become more common in older mice (Flurkey et al., 2007). All experiments described below were reviewed and approved by a local animal committee (University Health Network Animal Use Protocol #986.40) in accordance with the Guidelines of the Canadian Council on Animal Care.

### Electrode implantation

**FIGURE 1:**
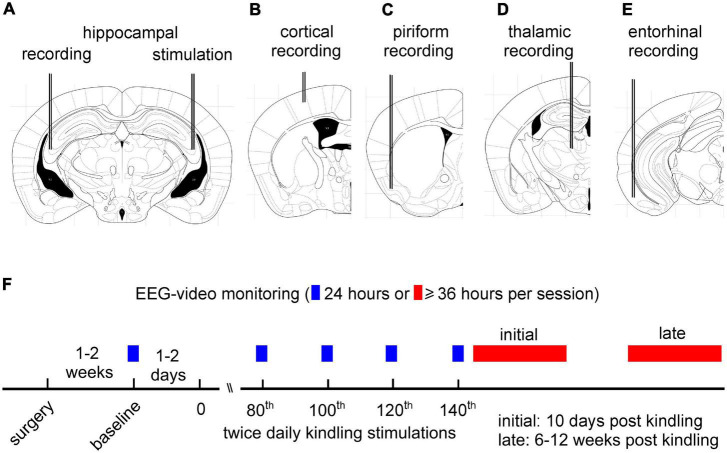
Schematic layout of intracranial electrode implantation and experimental design. **(A–E)** Schematic presentations of implanted intracranial electrodes in five groups of mice. Each mouse received implantation of two pairs of bipolar electrodes. The first electrode pair was placed at the hippocampal CA3 region to deliver kindling stimuli and obtain local recordings. The second electrode pair was positioned at unstimulated hippocampal CA3 **(A)**, parietal cortex **(B)**, piriform cortex **(C)**, dorsomedial thalamus **(D)**, or entorhinal cortex **(E)** for electroencephalography (EEG) recordings. **(F)** A schematic layout of extended kindling and EEG-video monitoring experiments. Hippocampal kindling stimulations were applied twice daily, and EEG-video monitoring for 24 h was conducted following 80, 100, 120, or 140 stimulations. Kindling was terminated if ≥2 spontaneous recurrent seizures (SRS) events were detected in the 24 h period. Continuous EEG-video monitoring for ≥36 h per session were performed within 10 days (initial period) or 6–12 weeks (late period) after termination of kindling stimulation.

Electrode construction and implantation were detailed previously (Bin et al., 2017; Song et al., 2018; Liu et al., 2019, 2021). All electrodes were made of polyimide-insulated and tip-exposed stainless-steel wires (outer diameter 0.125 mm; Plastics One, Roanoke, VA, USA). Each mouse was implanted with two pairs of twisted-wire bipolar electrodes. The first electrode pair was placed at the hippocampal CA3 region (bregma: -2.5 m, lateral: 3.0 mm, and depth: 3.0 mm; Franklin and Paxinos, 1997) to deliver kindling stimuli and obtain local recordings. The second electrode pair was positioned at one of five unstimulated ipsilateral or contralateral forebrain structures, including the hippocampal CA3, parietal cortex (bregma: -0.5 mm, lateral: 2.0 mm, and depth: 0.5 mm); piriform cortex (bregma: 0.5 mm, lateral: 3.0 mm, and depth: 5.0 mm); the contralateral dorsal-medial thalamus (bregma: -1.5 mm, lateral: 0.5 mm, and depth: 3.5 mm); and entorhinal cortex (bregma: -3.5 mm, lateral: 4.0 mm, and depth: 5.0 mm) (Figures 1A–E). These unstimulated structures were selected according to previous studies of extended kindling performed in monkeys and cats (Wada et al., 1974, 1975; Wada and Osawa, 1976; Hiyoshi et al., 1993). In following text, the five implantations were briefed as hippo-hippo, hippo-cort, hippo-pirif, hippo-tha, and hippo-ento, respectively. A reference electrode was positioned at a frontal area (bregma: +1.5 mm, lateral: 1.0 mm, and depth: 0.5 mm).

We used the above-mentioned implantation approach to explore regional EEG discharges in the kindled hippocampus and in an unstimulated forebrain area in individual mice while minimizing the potential complications associated with multi-electrode implantations in the small mouse brain. We stimulated the CA3 because it plays critical roles in genesis of hippocampal epileptiform activities (Le Duigou et al., 2014). In addition, the CA3 in the dorsal–middle hippocampus has a relatively wide distribution along the ventral–to–dorsal, lateral–to–medial and caudal–to–rostral planes hence convenient for electrode placement. In rats that underwent classic hippocampal kindling, the CA1 and dentate gyrus of the dorsal hippocampus were found to be different from ventral hippocampal counterparts regarding the threshold of evoked after-discharge and the number of stimuli for eliciting maximal motor seizure responses (Racine et al., 1977). As we stimulated the dorsal–middle CA3 region, the influences of similar regional differences on SRS development in kindled mice remain to be determined.

Overall, 116 mice were operated for electrode implantation. Of these implanted animals, 36 mice were euthanized within 48 h post-surgery due to bleeding, infection, poor physical conditions, or loss of implanted electrodes; 80 mice underwent baseline EEG-video monitoring 1–2 weeks after electrode implantation (Figure 1F).

### Hippocampal kindling

We used a train of stimuli (60 Hz for 2 s) for hippocampal kindling stimulation (Reddy and Rogawski, 2010; Jeffrey et al., 2014; Bin et al., 2017; Stover et al., 2017; Song et al., 2018; Liu et al., 2021). Constant current pulses (monophasic waveforms with pulse duration of 0.5 ms and current intensities up to 150 μA) were generated by a Grass stimulator (model S88) and delivered through a photoelectric isolation unit (model PSIU6; Grass Medical Instruments, Warwick, RI, USA). Initially, stimulations with incremental intensities were used to determine the threshold of evoked after-discharge in individual mice. Kindling stimulations were conducted at 25% above the threshold value. We attempted to keep stimulation intensity constant throughout the extended kindling period. However, the initial stimulation intensity often became inconsistent in evoking after-discharges, which might be largely due to contaminations or fouling of the implanted electrodes. Due to this complication, stronger stimuli at 60–110% above the initial threshold value were used if the initial stimulations failed to induce after-discharges in two consecutive days. Hippocampal kindling stimulations were applied twice daily, ≥5 h apart (Pinel and Rovner, 1978a,b; Milgram et al., 1995; Michalakis et al., 1998; Sayin et al., 2003; Brandt et al., 2004; Bin et al., 2017). Each stimulation episode lasted 2–3 min while the mouse stayed in a glass container for EEG-video monitoring.

Of the 80 mice that completed baseline monitoring, 70 mice underwent hippocampal kindling and 10 mice served as controls that experienced twice daily handlings for 60 days. Neither aberrant spikes nor SRS were observed from the control mice when they were monitored for 24 h after the 60 days handling manipulation. Extended hippocampal kindling induced SRS in 53/70 mice, including 12, 13, 11, 14, and 3 mice in the hippo-hippo, hippo-cort, hippo-pirif, hippo-tha, and hippo-ento groups, respectively. Three of the 53 mice died while being housed in home cages and other seven mice were mandatorily euthanized due to poor physical activities or severe skin infections. Hippocampal kindling was terminated in 17/80 mice after 23–96 stimuli due to loss/malfunction of implanted electrodes or health-related complications. Aberrant hippocampal spikes (Song et al., 2018), but not SRS, were observed from two of the 17 mice when monitored after 80 stimulations.

### EEG-video monitoring

Local differential recordings though the twisted-wire bipolar electrodes were used to monitor EEG signals. Signals were collected using two-channel or one-channel microelectrode amplifiers with extended head-stages (model 1,800 or 3,000, AM Systems; Sequim, WA, USA). These amplifiers were set with an input frequency band of 0.1–1,000 Hz and an amplification gain of 1,000. Amplifier output signals were digitized at 5,000 Hz (Digidata 1440A or 1550, Molecular Devices; Sunnyvale, CA, USA). Data acquisition, storage, and analyses were conducted using pClamp software (Version 10; Molecular Devices).

Continuous 24 h EEG-video monitoring was detailed previously (Bin et al., 2017). Each mouse was placed in a modified cage with food and water *ad libitum.* A slip ring commutator was mounted atop the cage and connected to the implanted electrodes *via* flexible cables for EEG recording. A webcam (model C615, Logitech, Canada) was placed near the cage to capture animal motor behaviors. Video recordings were acquired at 15 frames per second. Reflecting dim lighting *via* a 15-watt table lamp was provided to facilitate webcam monitoring during the light-off period. A cursor auto-click program (Mini Mouse Macro program)^1^ was used to manage concurrent EEG and video recordings and to save data every 2 h. EEG and video data were collected for approximately 24 h each day for up to seven consecutive days per session. As necessary, EEG-video monitoring was stopped for 10–30 min in the morning to allow for cage cleaning, addition of food and water, or other animal care procedures. EEG-video monitoring was performed over 24 h after 80, 100, 120, or 140 stimulations. Kindling stimulations were terminated if two or more SRS events were detected in the 24 h monitoring period. Continuous EEG-video for up to seven consecutive days were then performed within 10 days or 6–12 weeks after termination of kindling (Figure 1F).

### Brain histological experiments

Protocols for brain histology were detailed previously (Song et al., 2018; Liu et al., 2021). The control or extended kindled mice were euthanized for brain histology after the 60 days handling manipulation or the initial or late EEG-video monitoring (Figure 1F). Coronal brain sections (50 μm thick) were obtained *via* a Leica research cryostat (model CM3050) and stained with cresyl violet. Images were obtained using a slide scanner (Aperio digital pathology slide scanner AT2, Leica; at 20 × magnification) and analyzed using ImageScope (Leica) and Image J software (National Institute of Health, USA). Putative tip locations of the implanted electrodes were recognizable from three control mice and 10 extended kindled mice (Supplementary Figure 1).

### SRS detection

Spontaneous EEG discharges were recognized as repetitive spike waveforms with amplitudes ≥2-fold the background signal and durations lasting ≥10 s. Nearly all discharges displayed fast, low-voltage signals at the onset (Liu et al., 2021). Discharge termination, in most cases, featured a sudden cessation of spike activity and a subsequent signal suppression component lasting several seconds. Discharge durations were estimated as the time between the onset of fast, low-voltage signals and the cessation of spike activity. Discharge events were inspected independently by three researchers as described previously (Liu et al., 2021).

A modified Racine scale for mice (Racine, 1972; Reddy and Rogawski, 2010; Reddy and Mohan, 2011) was used to evaluate the severity of spontaneous motor seizures. Briefly, stage 0 indicated no response or behavioral arrest; stage 1 represented chewing or facial movement; stage 2 was characterized by chewing, head nodding, or unilateral forelimb clonus; stage 3 featured bilateral forelimb clonus; stage 4 was associated with rearing behavior; and stage 5 indicated falling over or loss of the righting reflex. Tail erection or Straub tail (Velíšková and Velíšek, 2017) was scored when the tail was elevated ≥90° perpendicular to the horizontal plane for ≥5 s. Spontaneous motor seizures were assessed independently by seven researchers through video reading as described previously (Liu et al., 2021). The concordance rates for recognizing stage 3–5 seizures were ≥90% among these researchers.

Occurrence of SRS was detected by combined EEG and video inspections. EEG signals were first screened to detect spontaneous discharges. Detected discharge events were time-stamped, and the corresponding video data were reviewed to score motor seizures. We used this approach for convenient SRS detection because spontaneous discharges with large amplitudes and co-expression in both stimulated and unstimulated sites were clearly distinguishable from background signals in our EEG recordings (Liu et al., 2021). In contrast, the scoring of motor seizures through video reading is laborious and often complicated by the mouse’s position within the cage or by the surrounding bedding material. Due to these complications, we did not analyze motor seizures by video reading alone.

In the present study, SRS with recognizable EEG discharges and accompanying motor seizures were analyzed; SRS without appreciable motor seizure activity were not included in the present analysis. The latter was due to errors in video acquisition or difficulties in video reading. SRS observed in individual mice within 10 days (initial period) or 6–12 weeks (late period) after termination of kindling stimulation were analyzed to explore potential seizure progression. In both the initial and late monitoring periods, SRS detected by continuous EEG-video monitoring for 24–112 h per session per mouse were analyzed. To avoid the influence of acute perturbations related to EEG electrode connection and cage changes on SRS occurrence, SRS detected in monitoring periods of ≤24 h were not included in the present analysis.

### Assessment of seizure-preceding behaviors

We used a modified approach from previous studies evaluating kainic acid- or pilocarpine-induced SRS in rats or mice (Hellier and Dudek, 1999; Pitsch et al., 2017). Seizure-preceding behaviors were assessed over a 3 min period prior to seizure onset and categorized as active or inactive. The mouse was considered to be active if it showed evident volitional movements that included walking, exploring the surroundings with rearing and/or head movement, eating, drinking, digging, grooming, and scratching its head or body. The mouse was considered to be inactive if it was immobile, standing still, or asleep without evident volitional movements. If the mouse showed mixed behaviors in the 3 min period prior to seizure occurrence, a score was made based on its behaviors in the 30 s period prior to seizure onset (Hellier and Dudek, 1999; Pitsch et al., 2017). Seizure-preceding behaviors were analyzed independently by three researchers (AZ, YS, and NA).

### Statistical analysis

Origin software (Northampton, MA, USA) was used for statistical tests. For normally distributed data, group differences were assessed using a Student’s *t*-test or one-way analysis of variance (ANOVA) followed by a *post-hoc* (Bonferroni) test. When data were not distributed normally, the Mann–Whitney U test or non-parametric rank-based ANOVA (Kruskal–Wallis) with a *post-hoc* (Dunn) test was used for group comparisons. A Chi-square test was used for rate comparisons. Data were presented as the mean and the standard error of the mean (SEM) throughout the text and figures. Statistical significance was set to *p* < 0.05.

## Results

**FIGURE 2:**
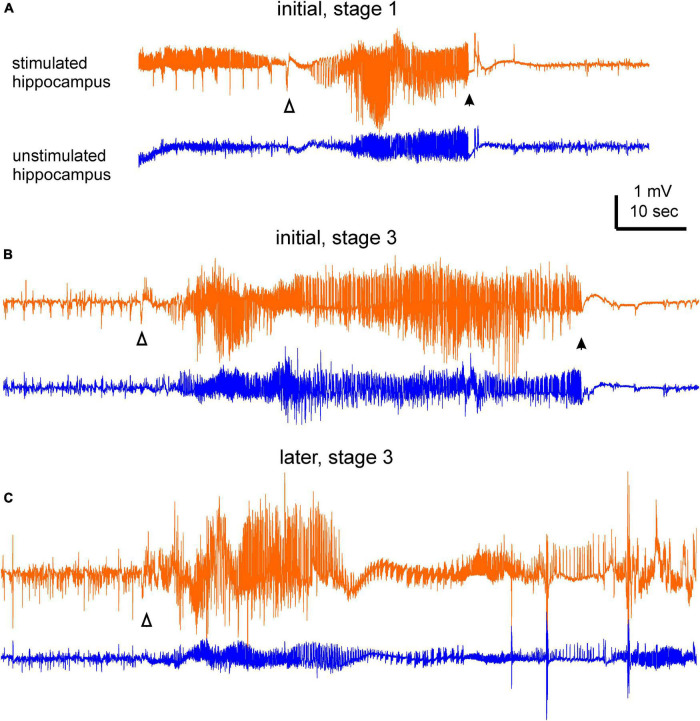
Examples of electroencephalography (EEG) ictal discharges. Bilateral hippocampal discharges were collected from a mouse during the initial and late monitoring periods. Discharges in **(A)** were associated with a stage 1 motor seizure, and discharges in **(B,C)** were associated with stage 3 motor seizures. Discharge onset and termination were denoted by open and filled triangles, respectively. Note in **(A)** there were spike activities prior to the discharge onset of the stimulated hippocampus. In **(C)** discharge termination was difficult to determine due to post-ictal spikes and artifacts. Motor seizures corresponding to these discharges were presented in the part 2 of Supplementary Videos 1–3.

Data were collected from mice in the five implantation groups (Figures 1A–E). Each mouse was implanted with two pairs of bipolar electrodes, one pair placed in the hippocampal CA3 for kindling stimulation and local recordings and another at an ipsilateral or contralateral unstimulated forebrain structure. Protocols for extended hippocampal kindling and SRS monitoring are schematically depicted in Figure 1F. SRS with recognizable EEG discharges and accompanying motor seizures were analyzed. Examples of EEG discharges and corresponding motor seizures are shown in Figures 2A–C and Supplementary Videos 1–3.

### Motor seizure scores

We first analyzed SRS that were detected within 10 days after termination of kindling stimulation (initial monitoring period). A total of 695 SRS events from 35 mice were analyzed in the present study. These included 77 events from four mice in the hippo-hippo group, 205 events from 11 mice in the hippo-cort group, 205 events from 10 mice in the hippo-pirif group, 162 events from seven mice in the hippo-tha group, and 46 events from three mice in the hippo-entor group.

Motor seizure severities were scored using the modified Racine scale (Racine, 1972) for mice (Reddy and Rogawski, 2010; Reddy and Mohan, 2011). Briefly, stage 1 and 2 motor seizures were recognized by chewing/facial twitch and head nodding with or without unilateral forelimb clonus; stage 3, 4, and 5 motor seizures were recognized by bilateral forelimb clonus, rearing, and falling. Spontaneous motor seizures could start with a brief period of behavioral arrest, followed by chewing/facial twitch (stage 1) and/or head nodding (stage 2) (Supplementary Video 1). These mild seizure behaviors could progress to bilateral forelimb clonus (stage 3; Supplementary Videos 2, 3). Rearing (stage 4) often followed bilateral forelimb clonus and persisted with or without forelimb clonus. Falling over or loss of the righting reflex (stage 5) was usually brief but could last several seconds, particularly when detected in the late monitoring period (i.e., within 6–12 weeks after kindling cessation).

Backward body movements were frequently associated with stage 3, 4, or 5 seizures, and occasionally with stage 1–2, motor seizures. Excessive salivation, recognized during seizures in patients with temporal lobe epilepsy (Shah et al., 2006) and in extended kindled monkeys and cats (Wada et al., 1975; Hiyoshi et al., 1993), were often observed in association with stage 3, 4, or 5 seizures. Tail erection (Straub tail), a convulsive behavior recognized in other rodent models of epilepsy/seizures (Henshall, 2017; Velíšková and Velíšek, 2017) but under-documented in extended kindled rats, was frequently observed in association with stage 3–5 motor seizures (87% of cases) but not with stage 1 or 2 motor seizures.

**FIGURE 3:**
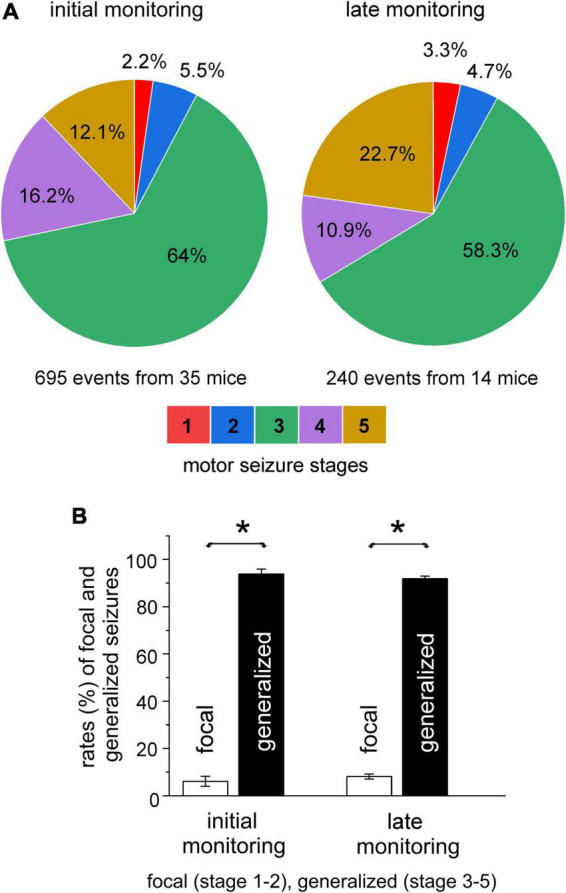
Severities of spontaneous motor seizures. Spontaneous recurrent seizures (SRS) were detected within 10 days or 6–12 weeks (initial or late monitoring period) after termination of kindling stimulation. Data were collected from mice of the five implantation groups. Motor seizure severities were scored using the modified five racine stages. **(A)** Summed proportions of motor seizure stages in the initial (left) and late (right) monitoring periods. **(B)** Summed rates of focal (stage 1–2) and generalized (stage 3–5) motor seizures for the two monitoring periods. Note that the rates of generalized or focal seizures were not significantly different between the initial and late monitoring period but rates of generalized seizures were significantly greater than those of focal seizures in both the monitoring periods (*p ≤ 0.05, one-way ANOVA).

The stages of spontaneous motor seizures were summed for mice in each implantation group. Stage 3 motor seizures were most frequently observed and accounted for 56–77% of all spontaneous motor seizures in the five groups of mice. Distributions of motor seizure stages were largely similar in the five implantation groups, except for the lack of detected stage 1–2 motor seizures in the hippo-entor group. When motor seizure stages from the five mouse groups were pooled together, stage 3 motor seizures made up 64% of total seizure events analyzed (Figure 3A–left).

Stage 1–2 and 3–5 motor seizures are generally considered as focal and secondary generalized seizure events, respectively (Sutula and Kotloski, 2017). We similarly categorized spontaneous motor seizures in our model. Focal and generalized motor seizures were estimated to represent, respectively, 7 and 93% of total SRS events for mice in the hippo-hippo group, 13 and 87% for mice in the hippo-cort group, and 5 and 95% for mice in either the hippo-pirif or hippo-tha group. The rates of focal and generalized motor seizures were not significantly different among the hippo-hippo, hippo-pirif, and hippo-tha groups when compared with each other (*p* ≥ 0.551, Chi-square test), but focal motor seizures were significantly more frequent in the hippo-cort group relative to the hippo-hippo, hippo-pirif, or hippo-tha group (*p* = 0.048, Chi-square test). The latter was largely due to 19 focal seizure events observed from a mouse in the hippo-cort group. When estimations for the five groups of mice were pooled together, focal and generalized seizures accounted for 7.7 and 92.3% of the total SRS analyzed (695 events from 35 mice; Figure 3B–left).

We next analyzed SRS that were detected 6–12 weeks after termination of kindling stimulation (late monitoring period). A total of 240 SRS events from 14 mice were examined, including 64 events from three mice in the hippo-hippo group, 119 events from five mice in the hippo-cort group, 39 events from four mice in the hippo-pirif group, and 18 events from two mice in the hippo-tha group. Spontaneous motor seizures detected in these 14 mice were pooled together, and rates of motor seizure stages were summed. Overall, motor seizure manifestations in the late monitoring period largely reproduced those occurring in the initial period. Stage 3 motor seizures remained the dormant convulsive behavior and accounted for 58.3% of total seizure events (Figure 3A–right). Tail erection was also observed frequently during stage 3–5 motor seizures (in 95% of cases), but only once during a stage 2 motor seizure. The rate of stage 5 motor seizures appeared to be greater in the late (22.7%) than in the initial (12.1%) monitoring period. However, when motor seizures were grouped as generalized and focal events, the rates of focal or generalized events were not significantly different between the two monitoring periods (8.1 vs. 7.7% and 91.9 vs. 92.3%; *p* = 0.788, Chi-square test); but there were significantly greater rates for generalized seizures than for focal seizures (*p* < 0.05, F value 771.08, T values ranged from -33.70 to 36.58, one-way ANOVA; Figure 3B–Right). For all analyzed motor seizure events, discharge durations in the stimulated hippocampus were comparable in the initial and later monitoring periods (47.1 ± 0.6 vs. 46.6 ± 1.0 s, *p* = 0.352, Mann–Whitney U test).

### SRS occurrences during the light-on and light-off periods

**FIGURE 4:**
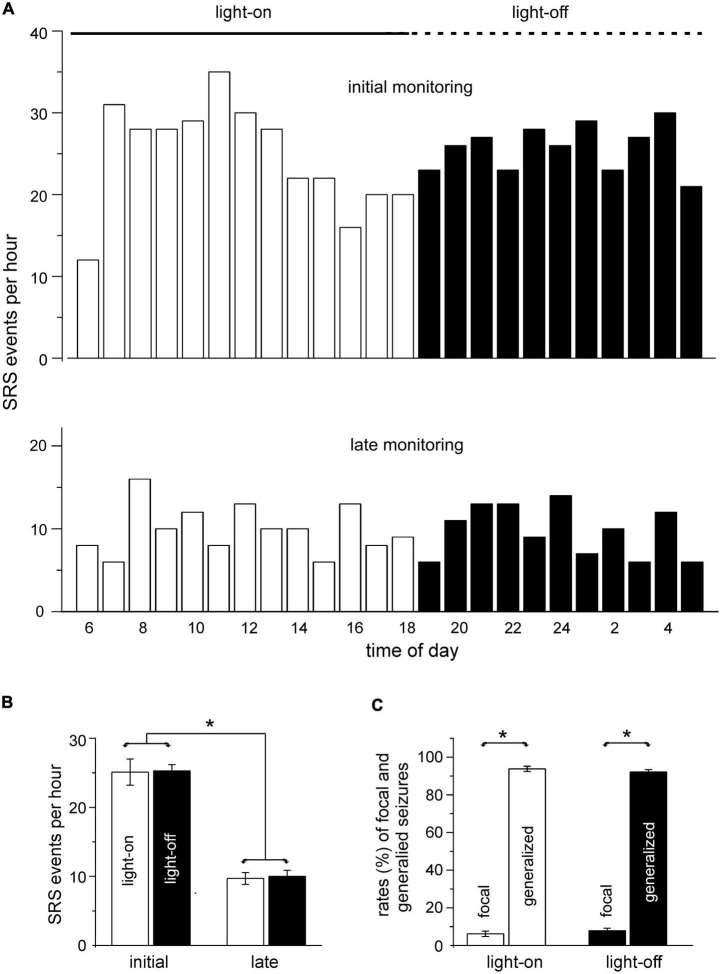
Spontaneous recurrent seizures (SRS) observed during the light-on and light-off periods. Data were collected from mice of the five implantation groups. SRS were detected within 10 days (initial monitoring period; 695 events from 35 mice) or 6–12 weeks (later monitoring period; 240 events from 14 mice) after termination of kindling stimulation. **(A)** Summed SRS incidence per hour plotted against times (24 h) of day. The light on or light off periods indicated by a solid or dotted line above the graph. **(B)** Mean seizure incidences estimated for the light on and light off periods. Seizure incidences between the light on and light off periods were not significantly different (*p* = 0.93 or 0.788) in both monitoring periods, but overall seizure incidences were significantly lower in the late than in the initial monitoring period (*p* < 0.001, Student’s *t*-test). **(C)** Overall rates of focal and generalized seizures estimated for the initial and late monitoring periods. Note that the rates of generalized or focal seizures were similar between the light on and light off periods and that there were greater rates for generalized seizures relative to focal seizures in both the initial and later monitoring periods (*p ≤ 0.05, one-way ANOVA).

In our experiments, mice were maintained in a 12 h light on/off cycle (light-on starting at 6:00 a.m.) while SRS were monitored *via* continuous EEG-video. To explore potential influences of the light on/off cycle on SRS occurrence, we sorted SRS events according to their occurrence times during the 24 h of the day and estimated the total number of SRS events per hour for the five groups of mice. Data collected in the initial monitoring period are presented in Figure 4A (top panel). Overall, SRS occurrences appeared to be more variable in the light on than in the light off period, as both the lowest (12 events/hour) and the highest (35 events/hour) seizure incidence were recorded at 6 a.m. and 11 a.m., respectively. However, the mean values of hourly seizure rates were comparable between the light on and light off periods (25.1 ± 1.9 vs. 25.3 ± 0.9 events/hour, *p* = 0.93, Student’s *t*-test; Figure 4B).

SRS occurrences in the late monitoring period were also variable during the 24 h of the day (Figure 4A, bottom panel). The mean values of hourly seizure rates were comparable between the light on and light off periods (9.7 ± 0.86 vs. 10.0 ± 0.88 events per hour; *p* = 0.788, Student’s *t*-test; Figure 4B). However, the overall mean SRS incidence per hour was significantly lower in the late than in the initial monitoring period (9.8 ± 0.6 events/hour vs. 25.1 ± 1.1 events/hour; p < 0.001, Student’s *t*-test; Figure 4B). To minimize potential perturbations associated with EEG electrode connections and cage changes on SRS occurrence, SRS detected in a period ≥ 24 h per recording session were further analyzed. In this way, SRS collected from only five mice were suitable for analysis for both the initial and late monitoring periods. Because most of the SRS were sampled from different mice in the two monitoring periods, individual and/or group variabilities in SRS incidence may partly explain the lower mean SRS incidences in the late period. Further experiments are needed to monitor a larger number of mice in both monitoring periods and to assess time-dependent SRS progression.

Overall, the rates of generalized seizures were significantly greater than those of focal seizures in both the light on and light off periods (92.1 vs. 7.9% and 93.8 vs. 6.2%; *p* ≤ 0.05), but there were no significant differences between the two periods with respect to the rates of generalized or focal seizures (*p* ≥ 0.05, F value 500.58, t values from -30.33 to 32.50, one-way ANOVA; Figure 4C).

### Seizure-preceding behavioral activities

We adopted an analysis approach as per previous studies in rodent models of kainic acid- or pilocarpine-induced SRS (Hellier and Dudek, 1999; Pitsch et al., 2017). Specifically, behavioral activities in a 3 min period before seizure onset were analyzed and categorized as active or inactive. Active seizure-preceding behaviors included walking, rearing and/or head bobbing, eating, drinking, digging, grooming, or scratching the head or body. Inactive seizure-preceding behaviors referred to immobility, standing still, or apparent sleep without evident volitional movement.

**FIGURE 5:**
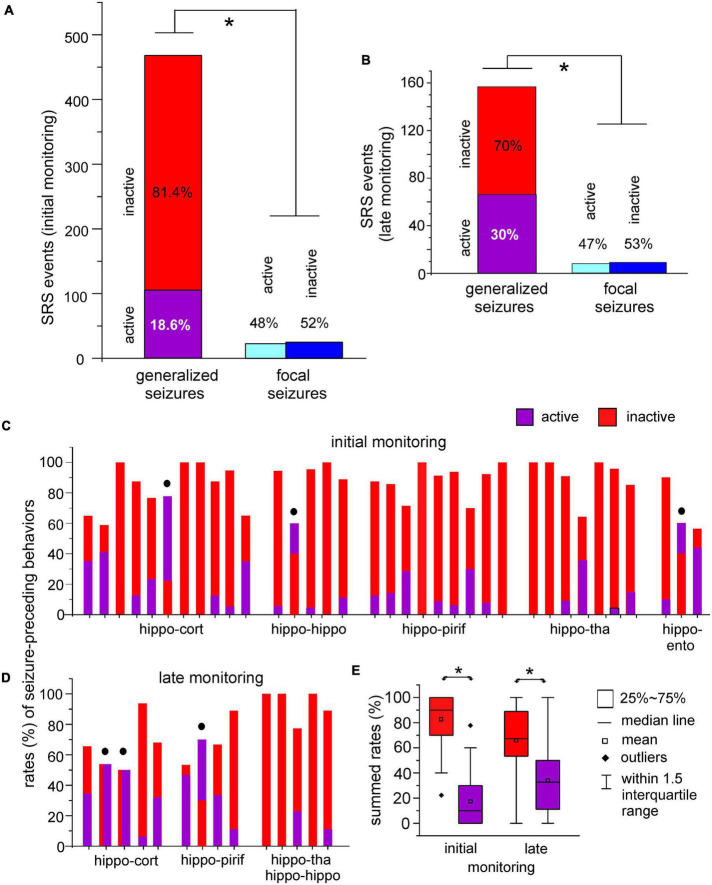
Seizure-preceding behavioral activities. **(A,B)** Spontaneous recurrent seizures (SRS) were observed within 10 days (initial monitoring period) and 6–12 weeks (late monitoring period) after termination of kindling stimulation. Data were collected from the five groups of mice. Inactive or active behaviors in a 3 min period prior to seizure onset were analysed. Spontaneous motor seizures were grouped as generalized (stage 3–5) and focal (stage 1–2) events. Seizure-preceding inactive and active behaviors were normalized as percentiles of total generalized or focal events analyzed. Rates of seizure-preceding inactive and active behaviors were significantly different between generalized and focal seizures (*p < 0.001 or *p* = 0.014, chi-square test). **(C,D)** Rates of active and inactive behaviors preceding generalized seizures in individual mice. Filled circles denoted mice with equal or greater rates of active relative to inactive behaviors. **(E)** Box plots showing summed rates of active and inactive seizure-preceding behaviors in initial and late monitoring periods. Rate of inactive behaviors were significantly greater than those of active behaviors (*p < 0.001 or *p* = 0.048).

For all generalized seizures collected in the initial monitoring period, 81.4 and 18.6% of events were preceded by inactive and active behaviors, respectively (Figure 5A–left). For all focal seizures collected in the initial monitoring period, 52 and 48% of events were, respectively preceded by inactive and active behaviors (Figure 5A–right). The rates of inactive and active behaviors preceding generalized seizures were significantly different for focal and generalized seizures (*p* < 0.001, Chi-square test).

Spontaneous recurrent seizure detected in the late monitoring period showed similar seizure-preceding behaviors. Generalized motor seizures were more frequently preceded by inactive than active behaviors (70 vs. 30%; Figure 5B–left), whereas focal seizures were almost equally preceded by either inactive or active behaviors (47 vs. 53%; Figure 5B–right). Rates of inactive and active behaviors preceding generalized and focal seizures were significantly different (*p* = 0.014, Chi-square test). For generalized seizures, active preceding behaviors showed an incremental trend in the late monitoring period (30%; Figure 5B–left) relative to the initial monitoring period (18.6%; Figure 5A–left), but such increase was not statistically significant (*p* = 0.071, Chi-square test).

We also analyzed seizure-preceding behaviors for individual mice in the two monitoring periods. Because focal motor seizures were not consistently observed for individual mice, rates of active and inactive behaviors preceding generalized motor seizures were estimated for individual mice (Figures 5C, D). While there were more inactive than active seizure-preceding behaviors in most mice, equal or greater rates of active relative to inactive behaviors were noticed for some mice (filled circles in Figures 5C, D). Summed rates of inactive preceding behaviors were greater than those of active preceding behaviors in the initial and late monitoring periods (Figure 5E; *P* < 0.001 or *p* = 0.048, Mann–Whitney U test or Student’s *t*-test).

## Discussion

Three main observations emerged from the present analysis. (1) SRS were found to predominantly manifest as generalized or stage 3–5 motor seizures. (2) SRS occurrences were not significantly altered by the light on/off cycle. (3) SRS with expression of generalized motor seizures were mainly preceded by inactive behaviors.

### Spontaneous motor seizures observed from rodent models of extended kindling

Spontaneous motor seizures with variable incidences and severities, or Racine stages, were recorded in individual rats following extended kindling (Pinel and Rovner, 1978a; Milgram et al., 1995; Michalakis et al., 1998; Brandt et al., 2004). For kindled rats under continuous 24 h EEG-video monitoring, generalized seizures and myoclonic jerks were observed in 36.4 and 13.6% of animals examined (Brandt et al., 2004). In our experiments, SRS in kindled mice were similarly monitored by continuous 24 h EEG-video (Liu et al., 2021). We found that stage 3 motor seizures were the main convulsive behavior in kindled mice, accounting for 64 and 58% of all observed SRS events in the initial and late monitoring periods, respectively. When spontaneous motor seizures were categorized as generalized (stage 3–5) and focal (stage 1–2) (Sutula and Kotloski, 2017), generalized seizures comprised about 92% of all SRS events analyzed. Overall, our present observations are not principally contradictory to the findings of Brandt et al. (2004) in kindled rats, since higher SRS incidence in kindled mice relative to kindled rats likely contributes to our frequent observations of generalized motor seizures.

Brief myoclonic jerks in association with EEG spikes were observed in kindled rats (Pinel and Rovner, 1978a; Brandt et al., 2004) but not in kindled mice. This discrepancy might be due at least partly to limitations in our experiments. Specifically, we used only one webcam to monitor each mouse and it was often difficult to recognize brief or mild convulsive behaviors when the mouse was not facing the webcam. In addition, we set the webcam at a low spatial resolution and acquired video data at 15 frames/second to facilitate video data storage. Moreover, video and EEG recordings were not synchronized. Thus, we might have missed the occurrence of myoclonic jerks in our analysis.

### Seizure-preceding behaviors in rodent models of temporal lobe epilepsy

Previous studies have induced SRS in rats or mice *via* application of pilocarpine, kainic acid, or intracranial electrical stimulation (Cavalheiro et al., 1991; Bertram and Cornett, 1994; Quigg et al., 1998, 2000; Arida et al., 1999; Goffin et al., 2007; Raedt et al., 2008; Tchekalarova et al., 2011; Pitsch et al., 2017). In these models, animals experienced acute status epilepticus and then exhibited chronic SRS after variable latency periods. While being monitored under experimental conditions with 12 h light on/off cycles, animals were found to exhibit SRS more frequently during the light on than during the light off period. We explored the potential influence of light on/off periods on SRS occurrence in extended kindled mice. Variable SRS occurrences were observed during the 24 h of the day, although mean SRS incidence was not significantly different between the light on and light off periods. While our present observations do not suggest a strong impact of the light on/off cycle on SRS occurrence, there were noteworthy confounds in our experiments. Specifically, EEG-video monitoring of SRS was not isolated from environmental noises, and dim lighting was used to facilitate video recording during the light off period. These suboptimal conditions might have influenced SRS occurrence and masked and/or altered the effects of the light on/off cycle. Therefore, caution should be taken when interpreting our study’s observations.

In rats or mice that exhibited SRS following kainic acid or pilocarpine application (Hellier and Dudek, 1999; Pitsch et al., 2017), seizure-preceding behaviors were determined in a 30 s period before seizure onset and categorized as active or inactive. SRS or generalized motor seizures were found to be preceded mainly by characteristic inactive behaviors. We adopted this analysis approach and assessed seizure-preceding behaviors in a 3 min period prior to seizure onset. We found that most of the generalized (stage 3–5) motor seizures were preceded by inactive behaviors, whereas focal (stage 1–2) motor seizures were nearly equally preceded by inactive or active behaviors. As generalized motor seizures comprised 92% of all SRS analyzed, our observations suggest that kindled mice exhibit SRS predominantly during inactive behaviors.

While our present data are in keeping with previous findings in rats or mice with kainic acid- or pilocarpine-induced SRS (Hellier and Dudek, 1999; Pitsch et al., 2017), our assessments of seizure-preceding behaviors were limited. Specifically, we were unable to distinguish sleep from wake immobility by video reading alone, as sleep-associated head-down position and eye closure were often difficult to determine if the mouse was not facing the camera. Additionally, in mice with electrodes implanted in the unstimulated cortex, the analysis of cortical slow waves often comprised by noises and/or interictal spikes. Future experiments with electromyography together with cortical EEG and video monitoring may help characterize the influence of the sleep-arousal cycle on SRS occurrence in our model.

### Potential mechanisms for the genesis of SRS in kindled mice

Our lab has detailed electrographic features of SRS in hippocampal-kindled mice (Liu et al., 2021). Spontaneous EEG discharges always concurred in the stimulated hippocampus and unstimulated structure, and nearly all discharges, regardless of their regional expressions, began with fast, low-voltage signals. Additionally, although discharge durations corresponding to focal seizures were noticeably variable, discharges corresponding to focal (stage 1–2) or generalized (stage 3–5) motor seizures were largely comparable in their waveforms and durations. Based on these observations, it is hypothesized that whereas epileptic activity involving multiple forebrain structures may initiate EEG discharges and associated motor seizures, forebrain EEG discharges may not directly control the severity of motor seizures (Liu et al., 2021).

An issue relevant to the above hypothesis is whether different brain structures are involved in the genesis of focal and generalized motor seizures in mice subjected to extended hippocampal kindling. Previous findings in standardly kindled rats have suggested that deeper subcortical structures are involved in the genesis and/or modulation of evoked generalized seizures (McNamara, 1986; Bertram and Cornett, 1994; Nolte et al., 2006). In the present analysis, tail erection or Straub tail was observed during most generalized motor seizures, but not during focal seizures. Tail erection has been recognized in other rodent models of epilepsy/seizures, and deeper subcortical structures, including the brainstem, are implicated in the genesis of this convulsive behavior (Velíšková and Velíšek, 2017). Thus, both previous findings and our present observations raise the possibility that the genesis of spontaneous generalized motor seizures in kindled mice may involve deeper subcortical structures. Further experiments that monitor and/or manipulate the activities of deeper subcortical structures (McNamara, 1986; Bertram and Cornett, 1994; Nolte et al., 2006) may provide valuable insights regarding genesis of SRS in our model.

Hippocampal neuronal injuries and/or structural alterations of varied degrees have been observed in rat models of classic kindling (Cavazos et al., 1994; Bengzon et al., 1997; Pretel et al., 1997; Danzer et al., 2010; Singh et al., 2013). In extended kindled rats that experience 150 or 100 evoked stage 5 motor seizures, hippocampal principal neurons were decreased to 40–49% of controls (Cavazos et al., 1994); cholecystokinin-positive GABAergic interneurons in the dentate gyrus were reduced by 25–76% as compared to controls (Sayin et al., 2003; but see Brandt et al., 2004). Previous works of our lab conducted basic histological assessments in a limited number of extended kindled mice with SRS. Gross brain injury independent of electrode implantation, such as structural deformation, cavity, and dark-stained scar tissues, were not observed in extended kindled mice (Song et al., 2018; Liu et al., 2021; Supplementary Figures 2A–C). Bilateral hemispheric areas were measured at eight coronal levels corresponding distances of 1.9, 1.2, 0.5, -0.2, -1.1, -1.5, -2.4, and -3.2 mm from bregma, respectively. The ratios of bilateral hemispheric areas were not significantly different between control mice and extended kindled mice (Supplementary Figure 2D), implicating no evident atrophy in the stimulated hemisphere. Whereas these observations are in general agreement with previous studies in extended kindled rats (Pinel and Rovner, 1978a; Milgram et al., 1995; Michalakis et al., 1998; Sayin et al., 2003; Brandt et al., 2004), brain/hippocampal cellular injuries as previously demonstrated in kindled rats (see references above) remain to be detailed in our model. In addition, prolonged implantation of intracranial electrodes in the small mouse brain likely caused local irritations, inflammatory reactions and/or tissue injuries as previously described in other models (Winslow and Tresco, 2010; Constant et al., 2012; Ravikumar et al., 2014). Moreover, monophasic current pulses we used for kindling stimulations might exacerbate these electrode implantation-related complications. It is conceivable that these injuries and complications might have impacts on SRS development and contribute to higher SRS incidences in our model relative to rat models of extended kindling (Pinel and Rovner, 1978a,b; Milgram et al., 1995; Michalakis et al., 1998; Sayin et al., 2003; Brandt et al., 2004). Further works are required to characterize these neuronal injuries and to determine their impacts on the incidence, regional involvement, and severity of SRS in our model.

## Summary

We provide original data detailing the severities of spontaneous motor seizures and seizure-preceding behaviors in a mouse model of extended hippocampal kindling. Despite limitations concerning our experimental setting and data analysis, our present observations may help further studies that use this mouse model for mechanistic and translational investigations. Our present data, together with the previous finding from rats or mice with kainic acid- or pilocarpine-induced SRS (Hellier and Dudek, 1999; Pitsch et al., 2017), support the hypothesis that ambient cage behaviors may be strong influencing factors of seizure occurrence in rodent models of temporal lobe epilepsy.

## Data availability statement

The original contributions presented in this study are included in the article/Supplementary material, further inquiries can be directed to the corresponding author.

## Ethics statement

The animal study was reviewed and approved by Animal Care Committee of University Health Network (Animal Use Protocol #986.42).

## Author contributions

AZ, YS, and NA performed the data analysis. LZ wrote the manuscript. NA edited the manuscript. All authors contributed to the article and approved the submitted version.

## Footnotes

1
http://www.turnssoft.com/mini-mouse-macro.html

## Supplementary material

Supplementary Videos Videos were collected from an extended kindled mouse during the initial and late monitoring periods, demonstrating a stage 1 (Video 1) and two stage three motor seizures (Videos 2, 3). Video segment corresponding to each motor seizure was divided into three parts that captured motor behaviors before, during and after seizure, respectively. Videos were arranged with each motor seizure appearing around the 5 s time stamps in the part 2 of Videos 1–3. Electroencephalography (EEG) discharges corresponding to these seizure events were illustrated in Figures 2A–C.
