# Supplementary material for: Convulsive behaviors of spontaneous recurrent seizures in a mouse model of extended hippocampal kindling
Source: Front Behav Neurosci. 2022 Dec 23;16:1076718. doi: 10.3389/fnbeh.2022.1076718 (PMC9816810; doi:10.3389/fnbeh.2022.1076718)
Supplement: Supplementary file 1 [file Data_Sheet_1.docx]

**
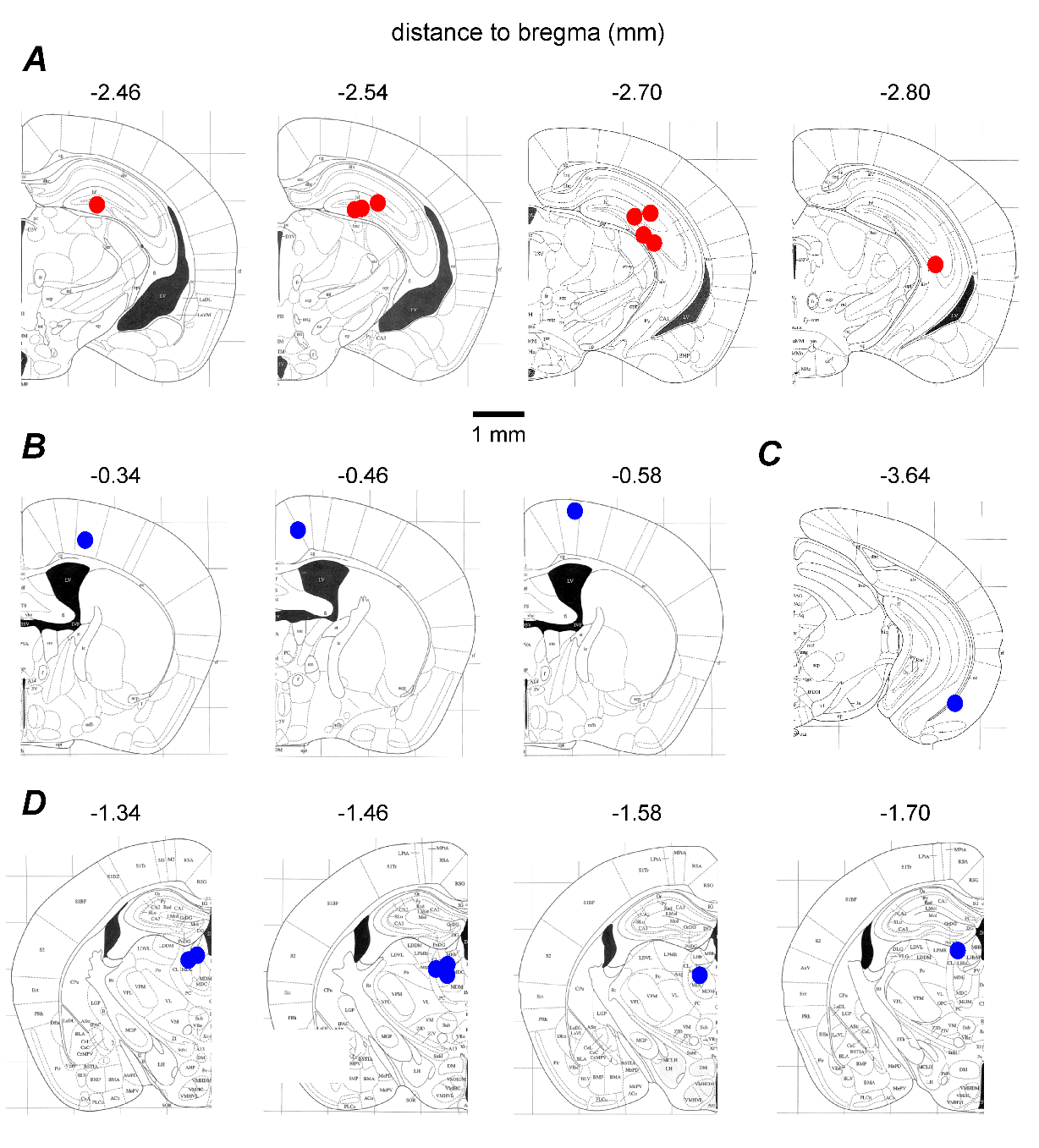
Supplementary Materials**

**Supplementary Figure 1. Histological examinations of implanted electrodes.** Data were collected from 10 extended kindled mice with SRS and 3 control mice. Putative tip locations of implanted electrodes were inspected in cresyl violet-stained coronal sections (50 µm thick). A-D, schematic presentations of coronal brain sections at indicated distances to bregma. Filled red or blue circles denoted putative tip locations in the stimulated hippocampus or unstimulated brain areas respectively. These locations appeared to be appropriate or near to the stereotaxic coordinates of targeted brain regions

**
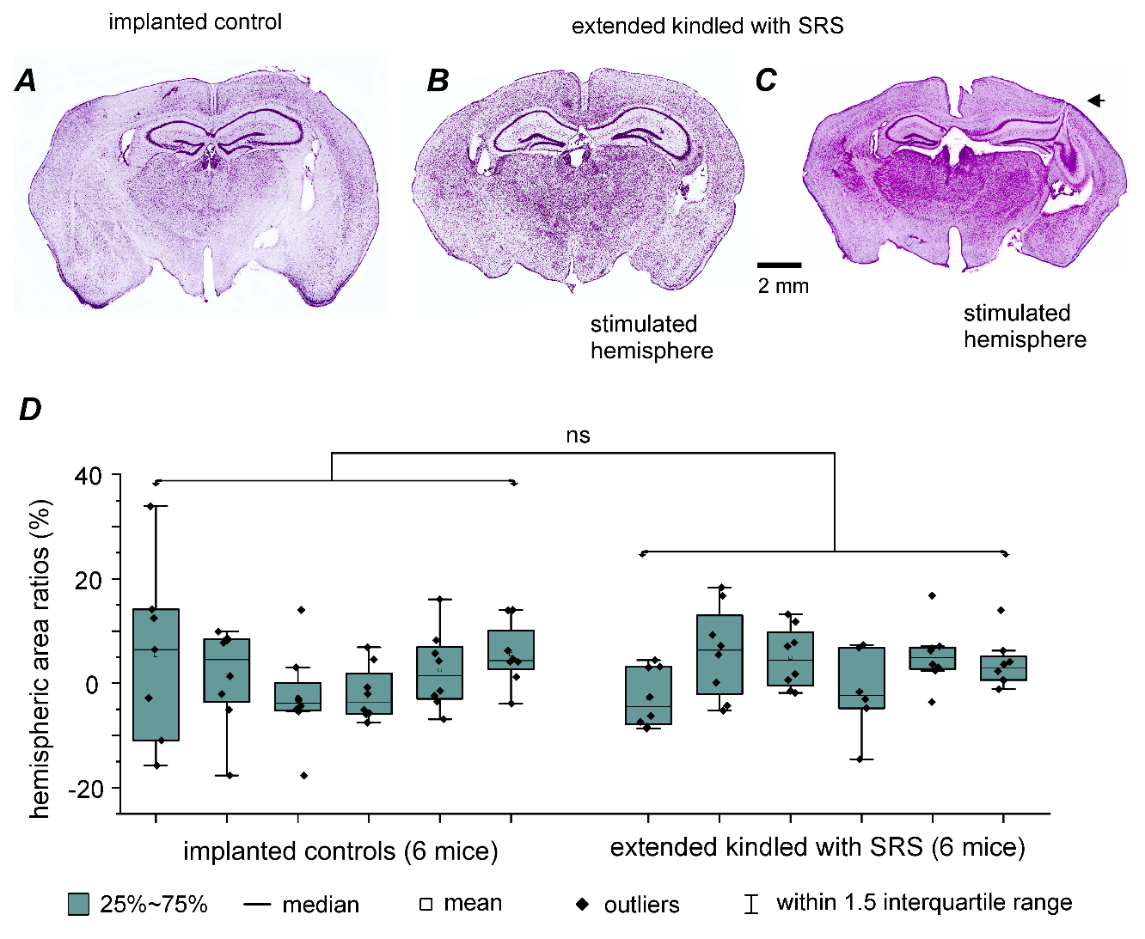
**

**Supplementary Figure 2. General histological observations from brain sections of control and extended kindled mice.** A-C, images of cresyl violet-stained coronal sections (50 μm thick) were obtained from a control mouse and two extended kindled mice with SRS. Disrupted hippocampal tissues in C (filled arrow) likely resulted from withdrawal of implanted hippocampal electrode during brain dissection. D, ratios (%) of bilateral hemispheric areas estimated for 6 control mice and 6 kindled mice with SRS. Data from individual mice were presented in box plots. Hemispheric areas were measured at eight coronal levels corresponding distances of 1.9, 1.2, 0.5, -0.2, -1.1, -1.5, -2.4 and -3.2 mm to bregma respectively. There were no significant (ns) group difference in these estimations (medium value comparison, p=0.79, Student’s t-test).
